# An RFID-Based Intelligent Vehicle Speed Controller Using Active Traffic Signals

**DOI:** 10.3390/s100605872

**Published:** 2010-06-09

**Authors:** Joshué Pérez, Fernando Seco, Vicente Milanés, Antonio Jiménez, Julio C. Díaz, Teresa de Pedro

**Affiliations:** Centro de Automática y Robótica, UPM-CSIC, 28500 Arganda del Rey, Madrid, Spain E-Mails: fernando.seco@car.upm-csic.es (F.S); vmilanes@iai.csic.es (V.M.); antonio.jimenez@car.upm-csic.es (A.J.); teresa.pedro@car.upm-csic.es (T.D.P.)

**Keywords:** automotive sensors, sensorial fusion, RFID, autonomous vehicle

## Abstract

These days, mass-produced vehicles benefit from research on Intelligent Transportation System (ITS). One prime example of ITS is vehicle Cruise Control (CC), which allows it to maintain a pre-defined reference speed, to economize on fuel or energy consumption, to avoid speeding fines, or to focus all of the driver’s attention on the steering of the vehicle. However, achieving efficient Cruise Control is not easy in roads or urban streets where sudden changes of the speed limit can happen, due to the presence of unexpected obstacles or maintenance work, causing, in inattentive drivers, traffic accidents. In this communication we present a new Infrastructure to Vehicles (I2V) communication and control system for intelligent speed control, which is based upon Radio Frequency Identification (RFID) technology for identification of traffic signals on the road, and high accuracy vehicle speed measurement with a Hall effect-based sensor. A fuzzy logic controller, based on sensor fusion of the information provided by the I2V infrastructure, allows the efficient adaptation of the speed of the vehicle to the circumstances of the road. The performance of the system is checked empirically, with promising results.

## 1. Introduction

Road fatalities are a major concern in the developed world. Recent studies [1] show that a third of the number of fatal or serious accidents are associated with excessive or inappropriate speed, as well as changes in the roadway (like the presence of road-work or unexpected obstacles). Reduction of the number of accidents and mitigation of their consequences are a big concern for traffic authorities, the automotive industry and transport research groups. One important line of action consists in the use of advanced driver assistance systems (ADAS), which are acoustic, haptic or visual signals produced by the vehicle itself to communicate to the driver the possibility of a collision. These systems are somewhat available in commercial vehicles today, and future trends indicate that higher safety will be achieved by automatic driving controls and a growing number of sensors both on the road infrastructure and the vehicle itself [2].

A prime example of driver assistance systems is cruise control (CC), which has the capability of maintaining a constant user-preset speed [3], and its evolution, the adaptive cruise control (ACC), which adds to CC the capability of keeping a safe distance from the preceding vehicle [4]. A drawback of these systems is that they are not independently capable of distinguishing between straight and curved parts of the road, where the speed has to be lowered to avoid accidents. However, curve warning systems (CWS) have been recently developed that use a combination of global positioning systems (GPS) and digital maps obtained from a Geographical Information System (GIS), to assess threat levels for a driver approaching a curve too quickly [5]; likewise, intelligent speed assistance (ISA) systems warn the driver when the vehicle’s velocity is inappropriate, using GPS in combination with a digital road map containing information about the speed limits [6].

However useful, these systems are inoperative in case of unexpected road circumstances (like roadwork, road diversions, accidents, *etc*.), which would need the use of dynamically-generated digital maps. The key idea offered by this paper is to use Radio Frequency Identification (RFID) technology to tag the warning signals placed in the dangerous portions of the road. While artificial vision-based recognition of traffic signals might fail if visibility is poor (insufficient light, difficult weather conditions or blocking of the line of sight by preceding vehicles), RF signals might still be transmitted reliably.

In the last years, RFID technology has been gradually incorporated to commercial transportation systems. A well known example is the RFID-based highway toll collection systems which are now routinely employed in many countries, like the Telepass system in Italy or the Autopass system in Norway. Other uses include monitoring systems to avoid vehicle theft [7], access control to car parking or private areas [8], and embedding of RFID tags in license plates with specially coded IDs for automatic vehicle detection and identification [9]. Placement of RFID tags on the road lanes has been proposed in order to provide accurate vehicle localization in tunnels or downtown areas where GPS positioning might be unreliable [10]. In the work by Seo *et al.* [11], RFID tagging of cars is offered as an alternative to traffic data collection by inductive loops placed under the road surface. The information about the traffic collected by a network of RF readers is then used to regulate traffic at intersection or critical points in the city. The work by Sato *et al.* [12] describes an ADAS, where passive RFID tags are arranged in the road close to the position of real traffic signals. An antenna placed in the rear part of the car and close to the floor (since the maximum transmitting range of the tags is about 40 cm) permits reading of the information stored in the tag memory and conveys a visual or auditive message to the driver. Initial tests at low driving speeds (20 km/h) show good results.

The work described in this paper is a collaboration between AUTOPIA (Autonomous Vehicles Group) and LOPSI (Localization and Exploration for Intelligent Systems), both belonging to the Center for Automation and Robotics (CAR, UPM-CISC). The aim of the research is to build a sensor system for infrastructure to vehicle (I2V) communication, which can transmit the information provided by active signals placed on the road to adapt the vehicle’s speed and prevent collisions. By active signals we mean ordinary traffic signals that incorporate long-range active RFID tags with information stored into them. This information is collected in real time by RFID sensors placed onboard of the vehicle (an electric Citroën Berlingo), which we have modified to automatically change its speed to adapt to the circumstances of the road. In particular, we have implemented a fuzzy logic control algorithm acting on the longitudinal speed of the vehicle, with actuators which control the vehicle’s throttle and brake to reach and maintain a given target speed.

This paper in organized as follows. A description of the sensors installed in vehicle and infrastructure is provided in Section 2. This includes the RFID traffic identification tags and the placement of the detector readers in the vehicle; the differential Hall Effect sensor installed in the vehicle’s wheels for better longitudinal speed control and the DGPS (Differential GPS). Section 3 discusses the system architecture, covering sensor data fusion, decision and control stages, followed by an explanation of the Cruise Control (CC) algorithm based in fuzzy logic in Section 4. Experimental demonstrations of the system in a test circuit in our institute’s grounds are described in Section 5. The paper ends with a discussion of the results in Section 6.

## 2. Sensor Systems

**Figure 1.**
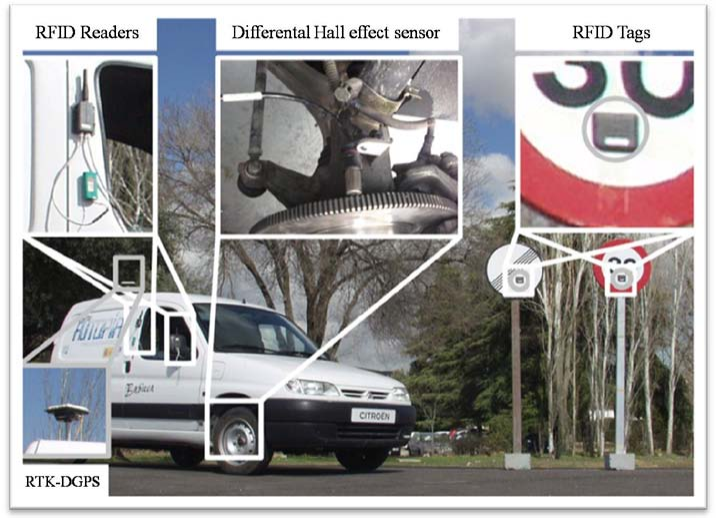
Sensors installed in the vehicle for the CC experiments: GPS unit, RFID readers and a speed measurement system based on a differential Hall Effect sensor mounted in a cogwheel (left-hand side). Traffic signals equipped with the RFID tags (right-hand side).

In this section we describe the sensors which have been installed in the vehicle (an electric Citroën Berlingo van) and the infrastructure in order to achieve intelligent speed control. The sensors subsystems are: an RFID-based system for traffic signal detection and identification (in the infrastructure and onboard the vehicle), a differential Hall effect sensor placed in the vehicle’s wheels for high accuracy speed measurement, and a differential global positioning system (DGPS) to locate the vehicle and to set the sampling frequency of our control loop. The physical arrangement of these sensors in the vehicle and the infrastructure is shown in Figure 1.

### 2.1. Radio Frequency Identification (RFID) Sensors

An RFID system consists in a set of emitters or tags which, periodically or upon interrogation, transmit a short digital radiofrequency message containing an identification code (unique to each tag) as well as some data stored in the tag’s memory. These data can be obtained remotely by a computer equipped with an RFID reader. Besides the tag ID, which confirms the presence of the tag within the detecting range of the reader, the RFID reader measures the received signal strength (RSSI) of the RF signal, which is an indicator of the range from tag to reader.

The main advantage of RFID systems—with respect to other RF technologies, which could be used for infrastructure-to-vehicle (I2V) communications—is its low cost and minimum infrastructure maintenance, which results in a high scalability and easy deployment of the infrastructure. The kind of active RFID tags used in this research are cheap (10–20 euros each), can be easily attached to the traffic signals and last for at least five years. The right side part of Figure 1 shows the RFID tags placed on the traffic signals.

For this application we have chosen RFID equipment provided by Wavetrend Inc. We use TG800 active tags, emitting identification signals regularly every 1.5 s at an RF carrier frequency of 433 MHz. These tags are rugged and are powered by their own batteries. Two model RX-201 RFID readers are placed on the right side of the computer controlled vehicle, and are polled by a PC through the serial port (two independent readers are used for redundancy, since occasionally tag detections might be missed by one reader). RFID data are transmitted upon detection through an Ethernet connection. It is convenient that the RF signals from the tags placed in the traffic signals are detected from a distance large enough that timely control actions might be taken over the car. Physically, the transmitting range of an RF system is limited by the interference of the wave transmitted directly from emitter to reader, and the one reflected by the ground plane [Rappaport 1996]. For ranges larger than a critical distance, these two waves cancel each other out, and the received signal strength decreases sharply. An approximation to the useful range of a RF transmitting system is given by:

(1) $$d_{T}={2\pi h}_{T}\;h_{R}/\lambda$$

where *λ* is the wavelength of the RF signal, and *h_T_* and h*_R_* are respectively the heights of the emitter and the receiver.

From Equation 1, it’s clearly convenient to place both reader and RFID tags as high above the ground as possible. In our case, tags are placed in the plate of the traffic signal, at 2.05 m above the ground, and the readers are situated in one side of the car, at 1.6 m and 1.4 m above the ground, as shown in Figure 1. For a wavelength of 0.69 m, this gives an approximate propagation distance of 28 m. Experimentally we found that signals were reliably detected 30 m away from the reader, and occasionally at even larger distances.

**Figure 2.**
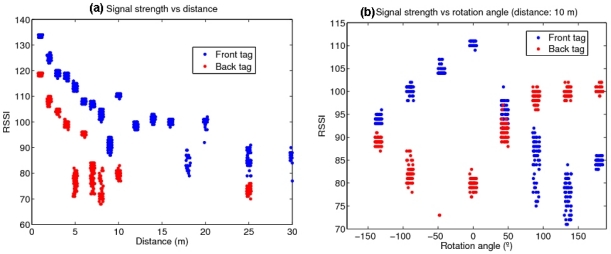
Signal strengths of tags placed in the front and back sides of the traffic signal, as (a) the reader is placed in front of the signal and its longitudinal distance is varied; (b) the reader is fixed at 10 m and the traffic signal is rotated through a complete turn.

Besides determining if a traffic signal is within a given range of the reader (placed in vehicle, as shown in the left-hand side of Figure 1), its orientation can be also important, since if the signal is facing away from the vehicle, it will convey no information for it, and only for the vehicles circulating the other way. To distinguish the signal orientation, one tag was placed in the front of the traffic signal and another in the back. Figure 2 shows the dependence of the received signal strength with (a) the distance of the traffic signal and the reader, and (b) the rotation angle of the signal. The shielding of the transmitted RF waves caused by the metallic plate of the signal greatly attenuates the RSSI readings of the tag from its back and permits in principle to determine its orientation.

**Figure 3.**
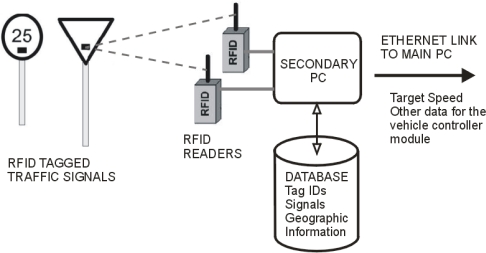
Operational block diagram of the RFID subsystem.

The operation of the RFID subsystem onboard the vehicle is described with the block diagram of Figure 3.

Ordinary traffic signals equipped with RFID tags transmit their identification code and are detected by the RFID readers onboard the vehicle. The information is transmitted to a PC, which determines the correspondence between tag IDs to traffic signals in a database (which may also contain geographic information about the area where the signals are situated). This secondary PC communicates the new target speed as well as other relevant data for the control of the vehicle to the main PC through an Ethernet connection.

### 2.2. Differential Hall Effect Sensor

One of the groups working in this research (AUTOPIA) has extensive experience in the development of control strategies which can be used in mass-produced cars to reproduce throttle and brake behaviour [4,14]. The control systems developed by our group are of two kinds: the first uses the Controller Area Network (CAN) bus which provides information from the on-board sensors, as the actual speed; the second uses the signal coming from the speedometer, but in this case the precision is limited to four pulses for one turn of the vehicle’s wheels, which gives insufficient resolution at low speeds. Both measurement methods depend on the quality of the speed sensor already installed in the vehicle, which may not satisfy the requirements for control of the longitudinal motion.

Therefore, in order to obtain a speed measurement sensor that guarantees high precision, that can be portable to any mass-produced vehicle (equipped with CAN bus or not), and supports high sampling frequency in the control loop, a new differential Hall Effect sensor was installed. The sensor was coupled to a cogwheel attached to one of the forward wheels of our car (see Figure 1). The output of the sensor is connected to the main PC using an analogue to digital converter, permitting us to obtain the vehicle’s speed easily. Specifically, the advantages of this new sensor are: more precision (directly related to the number of teeth in the cogwheel), faster sensor reading times, compatibility with standard devices (USB-analogue card) and, finally, the possibility of increasing the sample time of the longitudinal control.

Taking into account the available space, a cogwheel with a diameter of 266 mm and 180 teeth was used. So, the covered distance (d_c_) by the car is given by:

(2) $$d_{c}=2\pi r(\frac{\mathrm{pulses}}{180})$$

where *r* is the wheel radius that is determined experimentally and *pulses* is the number of pulses count by the Hall effect sensor. The CPU internal clock is used as base time to calculate the speed of the vehicle. The control cycle is set by the DGPS frequency—up to 10 Hz. The error in the control loop depends of the vehicle’s speed. In the trials described in this paper, the maximum speed is 30 km/h on straight segments due to limitations of our test track, therefore the maximum error is around 80 cm on the road. For higher speed (the maximum speed allowed in our electric car 90 km/h) this error can reach 2.5 m, which is small compared with human drivers. The minimum required frequency in order to select an appropriate differential Hall effect sensor is 2.6 kHz. The sensor selected for this application was the *Honeywell* SNDH-T4L-G01. It presents a good resolution and enough commutation frequency -from 0 to 15 kHz-. A National Instruments (NI USB-6008) card connected via USB to the laptop is in charge of receiving the pulses generated by the Hall effect sensor.

### 2.3. Real Time Kinematic-Differential Global Positioning System (RTK-DGPS)

The main sensor used for acquiring driving information is an on-board real time kinematic-differential global positioning system (RTK-DGPS)—Trimble’s MS750 system—that allow us to locate the vehicle with centimetric precision. The control loop time is 100 ms, given by frequency of the GPS (10 Hz). Since the autonomous guidance is out of the scope of this work, the RTK-DGPS is only used for evaluation of the performance of the intelligent speed controller (the experiments described in Section 5). In real life, a low-cost commercial GPS with ISA capabilities can be used instead with the proposed system.

## 3. Description of the Cruise Control Architecture

**Figure 4.**
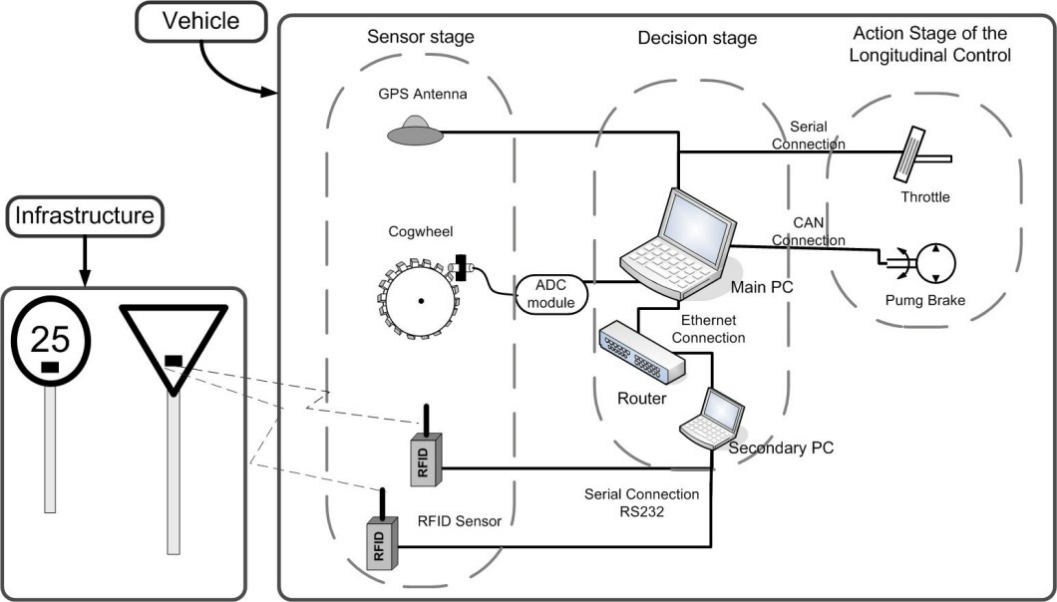
Control scheme onboard the vehicle and its interaction with the infrastructure.

The proposed architecture for cruise control is shown in Figure 4, and comprises two parts: placement of RFID sensors (tags) in the road’s traffic signals, and the on-board systems in the vehicle, which we will describe in this section.

The autonomous longitudinal control of the vehicle takes place in three stages: environment perception (sensor data acquisition), decision, and control action. The perception stage corresponds to the acquisition of information from the environment and the car itself, and passing it to the control computer. There are three sensorial inputs: RFID detections from active traffic signals detected on the road (this is performed by a secondary PC in the car and transmitted to the main computer by an Ethernet connection); an on-board GPS receiver for acquiring driving information; and, finally, readings from the Hall effect sensor located in one of vehicle′s forward wheels, with an accurate estimation of the vehicle’s velocity.

The decision stage is responsible for interpreting the data obtained in the perception phase, and is divided in two phases. The first is the co-pilot, whose mission is to select among all the different controllers. These controllers—all of them based on fuzzy logic—have been designed to take into account any possible traffic condition in the longitudinal control—straight-road tracking, bend tracking, intersections or adaptive cruise control [4,14]. The second phase is the pilot, made up by the low level controllers that decide which is the best controller for each traffic situation and generate the output for the actuators. This phase is divided in the lateral and longitudinal control, to evaluate the behaviour of the proposed system, only the fuzzy longitudinal controller is needed.

The latter stage is the actuation stage, which is in charge of the execution of the goals coming from the previous stages. Its function is to adapt the output value generated by the pilot to values that can be applied to the actuators, *i.e.*, throttle and brake pedals. The actuators have been modified to permit autonomous control of the longitudinal speed/position of the vehicle, but its lateral position is still controlled by the driver with the steering wheel.

### 3.1. Throttle Automation

The throttle is controlled with an analogue signal that represents the pressure on the pedal, generated with an I/O digital-analogue CAN card. A switch is used to commute between the original circuit of the throttle pedal and the autonomous system. We use a NI USB-6008 National Instrument card to decode the speed, directly from the Hall Effect sensor, establishing the analogical value corresponding to the desired level of pushing in the throttle pedal via an analogue CAN card.

### 3.2. Brake Automation

The brake action is the most critical, since it must be able to stop the car in case of a failure of the autonomous system. For robustness and safety, we mounted an electro-hydraulic braking system in parallel with the original one provided by the car manufacturer. Two shuttle valves are connected to the input of the braking system in order to keep the two circuits independent. Each valve permits flow from either of two inlet ports to a common outlet by means of a free-floating metal ball that shuttles back-and-forth according to the relative pressures at the two inlets. One of the inlets is connected to the electro-hydraulic braking system and the other to the original one. These valves permit the two braking systems to coexist, but independently of each other.

A pressure limiter tube set at 120 bars is installed in the system to avoid damage to the circuits. Two more valves were installed to control the system: a voltage-controlled electro-proportional pilot to regulate the applied pressure, and a spool directional valve to control the activation of the electro-hydraulic system by means of a digital signal. These two valves are controlled via the same I/O digital-analogue CAN card that the throttle.

## 4. Intelligent Speed Controller

When it comes to design a vehicle speed controller, the priority was to guarantee the car’s occupants comfort. The denomination *Comfort Driving* is an imprecise term and its limits can be established differently according to the situation. One commonly accepted threshold in the automotive sector is found in reference [15], which fixes the maximum acceleration for comfort at 2 m/s^2^.

Our control system is designed to obey the rules of the traffic code, and the reference speed should respect the maximum allowed speed for each road. This speed will be set up by ordinary traffic signals whose position is read from a digital map, and, in extraordinary circumstances, as described in the introduction, by the active traffic signals detected by RFID detectors on board of the vehicle. Once the potential risk has passed, the reference speed for the road is recovered.

Since Tagaki and Sugeno developed controllers based on fuzzy logic systems [16,17], many industrial processes are controlled using the knowledge from expert operators. The main advantage of a fuzzy controller is that an exact mathematical model of the system is unnecessary.

**Figure 5.**
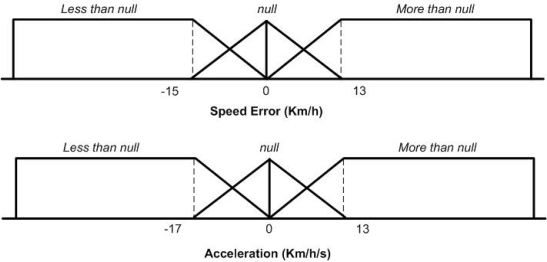
Longitudinal control input membership function: speed error and acceleration.

In order to carry out a good speed control, two inputs are used coming from the Hall Effect sensor: the speed error defined as the difference between the actual speed and the target speed—in km/h—and the acceleration—in Km/h/s (this is computed by differentiation of the vehicle’s speed) [14]. As output, the action over both pedals are defined as [−1,1], where −1 indicates the brake pedal is completely depressed (Down) and 1 indicates the maximum action is produced over the throttle (Up). Figure 5 shows the input membership function.

An experimental fuzzy coprocessor (ORBEX) was developed [18], which is an inference motor with a straightforward natural-language-based input language. ORBEX functions with Mamdani’s inference method, with singleton-type membership functions to codify the output variables. The fuzzy controller developed consists of a rule base containing expert knowledge and a set of variables representing the linguistic values considered. In spite of the highly nonlinear behaviour of vehicle, a human driver is capable of driving it based on his experience. We use as expert knowledge this intuitive human experience in order to design a controller capable of carrying out the vehicle’s speed control. The rules of this controller are as follows:

- **R1:    IF** *Speed Error* **MORE THAN** null **THEN** *Accelerator* up
- **R2:    IF** *Speed Error* **LESS THAN** null **THEN** *Accelerator* down
- **R3:    IF** *Acceleration* **MORE THAN** null **THEN** *Accelerator* up
- **R4:    IF** *Acceleration* **LESS THAN** null **THEN** *Accelerator* down
- **R5:    IF** *Speed Error* **MORE THAN** null **THEN** *Brake* down
- **R6:    IF** *Speed Error* **LESS THAN** null **THEN** *brake* up
- **R7:    IF** *Acceleration* **LESS THAN** null **THEN** *brake* up

The Speed Error is the proportional component of the control and the Acceleration is the derivative component. This means that, when the speed of the car is not at the desired value, the Speed Error adjusts the throttle pressure, and the Acceleration smoothes out the actuation of this command [14]. In that way, rule R1 acts when the current speed is lower than the preset CC speed and works cooperatively with the brake rule R5. The second rule R2 is the complementary rule and interacts with the second brake rule (R6). The last rule (R7) corresponds to the derivative part of the control system, smoothing the speed adaptation manoeuvres and actuating cooperatively with the throttle rule R4.

## 5. Experimental Results

This section describes empirical tests in a private driving circuit in order to validate the speed control architecture proposed in this paper. Two experiments were performed: the first to determine the reliability of the detection of RFID tagged traffic signals from the vehicle moving at different speeds; and the second to effectively evaluate the intelligent speed control adaptation to the information on the circumstances of the road provided by the signals.

### 5.1. Test Environment

**Figure 6.**
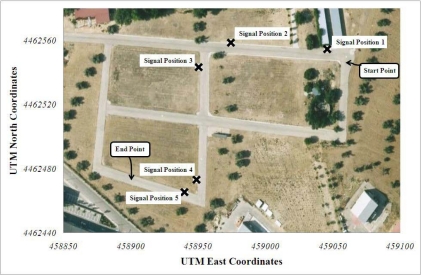
Test circuit in the CAR facilities showing the traffic signal positions for the intelligent Cruise Control using RFID experiment.

The experimental tests have been performed in a private driving circuit in the Centre of Automation and Robotics (CAR) of the Spanish National Research Council and Polytechnic University of Madrid (CSIC-UPM) facilities. A simple setup to demonstrate the validity of RFID-based automatic vehicle speed control was arranged, with the circuit and the position of traffic signals shown in the aerial view of Figure 6. Each signal was configured to convey different target speeds to the vehicle’s driver (five signals were used for the tests), as described below. The data of the RFID-tagged traffic signals is detailed in table 1. RFID tags were attached to the front and the back parts of the traffic signal’s plate at a height of 2.05 m over the ground, while the readers were in one side of the car, at 1.6 m and 1.4 m of height.

### 5.2. Tag Detection Experiments

A simple experiment was performed to check how reliably a traffic signal could be detected from a moving vehicle. The car, equipped with two readers on its right side, circulated close to a traffic signal placed in one intersection of the CAR circuit, at three different speeds (6, 12 and 24 km/h). Tag detections were recorded by the onboard computer, while the position of the vehicle was determined with high accuracy by the car’s DGPS system.

**Figure 7.**
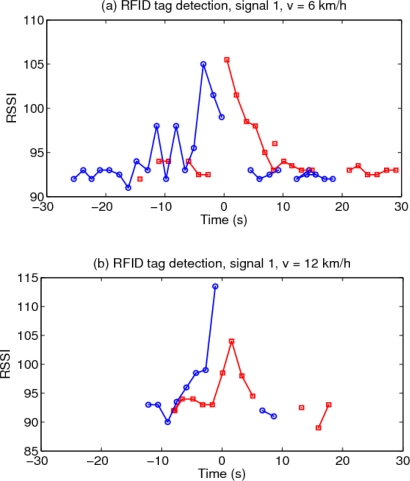

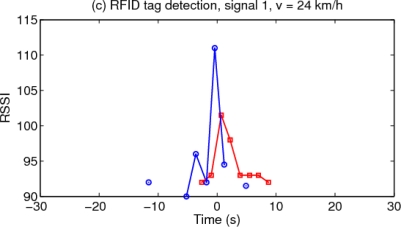
Readings of front (blue) and back (red) tags of the first traffic signal detected by the car at three different speeds (6, 12, and 24 km/h)

Figure 7 shows the results of the tag activity, with the origin of the time axis chosen at the moment when the car passes right by the traffic signal.

**Figure 8.**
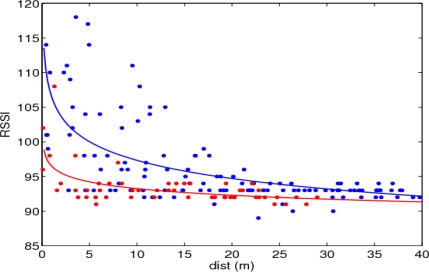
Statistical distribution of signal strength readings *versus* distance, for tags in front (blue) and behind (red) the traffic signal; and best fits produced with a path-loss model for signal strength.

The statistical distribution of the tag’s signal strength *versus* distance, for tags in front and behind the traffic signal, obtained from the collected data of the trajectories of Figure 7, is shown in Figure 8. It is seen that RSSI values decrease with increasing distance to the signal, and that the tags behind the body of the signal are detected only at lower ranges, and with correspondingly lower signal strengths. However, any individual reading value is not predictable due to the high measurement variance. A path-loss model of the standard form: *RSSI* = *RSSI*_0_ − *α*log *d*, fitted to the data is also shown.

### 5.3. Autonomous Behaviour

To test the control system proposed, a commercial vehicle—a Citroën Berlingo van with electric-powered motor—was instrumented to allow the automatic control of the vehicle’s actuators (Figure 1). The vehicle’s automatic control was designed in a way such that the longitudinal and lateral control blocks are independent [19].

**Figure 9.**
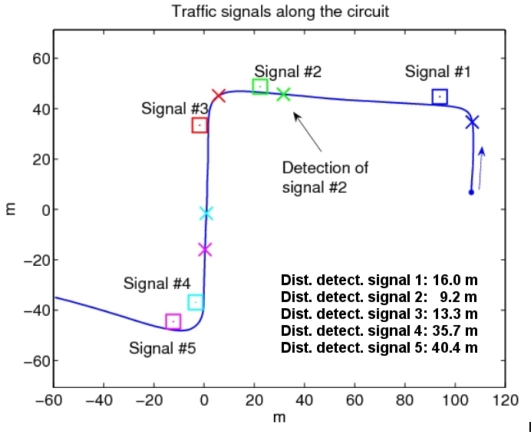
Circuit for vehicle control experiments and positions (□) of the five speed limit signals. The X marks indicate the positions in the road where the corresponding traffic signals are first detected by the RFID readers on the vehicle.

The test consists in traversing the circuit of the Figure 9, adjusting the speed of the vehicle automatically attending to the information provided by the traffic signals equipped with RFID tags. The vehicle’s speed is kept at a normal value—as indicated by signal 1 (Figure 9)—in straight portions of the road until detection of curve warning signals—signals 2 and 4 (Figure 9)—which indicate the proximity of a sharp bending curve, for which the driver is required to reduce the vehicle’s speed. Signals 3 and 5, placed at the exit of the curves, indicate that the vehicle can accelerate again up to the normal speed limit of the road.

**Figure 10.**
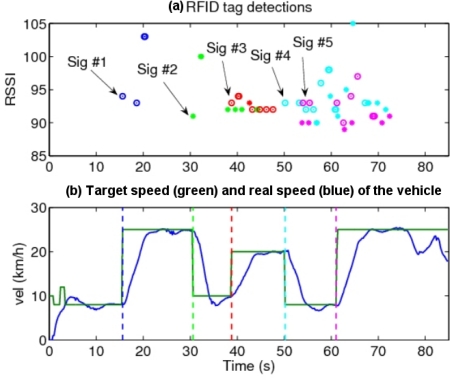
(a) Detection of the RFID tags of the traffic signals of Figure 6; (b) change of the target speed (green) and real speed (blue) of the vehicle according to the RFID information.

Figure 10 shows the RFID readings while the vehicle traverses the circuit, which consist of discrete detections of tags, their associated tag IDs, and the received signal strength values (RSSI). The RSSI is roughly correlated with the distance to the signal, although an exact relationship cannot be used, in view of the results of Figures 7 and 8. On average, traffic signals were first detected from a distance of 23 m (minimum was 9 m for signal 2, and maximum was 40 m for signal 5, see the data in Figure 9). As it can be seen, sometimes tag detections corresponding to consecutive traffic signals occur almost simultaneously, if they are within the detecting range of the RFID readers.

The vehicle’s control algorithm changes the speed setting of the vehicle upon the first detection of the RFID tag associated with a given traffic signal according to the information conveyed by it, then maintains the target velocity until detection of the next signal. For the case where traffic signals are placed too close (for example, signals 4 and 5 at the beginning and end of the second curve), tag detections might be produced almost immediately; in this case the computer includes a time delay for the change of the vehicle speed since the first detection of the first signal, that permits the vehicle to reach the part of the road affected by the second traffic signal. This delay depends on the measured speed of the vehicle, the separation of the adjacent traffic signals on the road (known from the database of Figure 3), and the speed with which the vehicle’s controller can actually change its velocity. It can be seen (Figure 10) that a rather simple control scheme produces good results.

## 6. Conclusions

This paper presents an architecture for automatic adaptation of the longitudinal speed control of a vehicle to the circumstances of the road which can help to decrease one of the major causes of fatalities: the excessive or inadequate vehicle speed. Our approach is based on a combination of three different sensor technologies: RFID tagging of traffic signals to convey their information to the car, Hall Effect sensors located in the vehicle’s wheels for high accuracy measurement of the speed of the car, and DGPS for precise positioning of the vehicle and control loop time. Sensor fusion is applied to the information received by these subsystems, and used to adjust the longitudinal speed of the vehicle with a fuzzy controller. The proposed on-board architecture is portable and easily adaptable to any commercial car with minimal modifications.

The system shows promising results, since active RFID technology permits to detect the presence and identity of the traffic signals reliably and sufficiently in advance, so corrective actions on the vehicle’s behaviour can be taken. In the empirical trials in our installations, the vehicle’s speed was successfully changed as a result of the detection of the signals, increasing the driver’s safety. The technology developed can assist human drivers in difficult road circumstances, as well as a complement ISA or CWS systems if the car is already equipped with them.

In our experiments, only the test vehicle was present on the road. In normal driving situations, we can expect other vehicles circulating nearby and possibly blocking or attenuating some of the RFID transmitting signals, especially with large vehicles like trucks. In this aspect, more experimentation is needed to know how this circumstance will affect the vehicle’s control performance. A possible solution is the use of redundant RFID tags (since their cost relatively low), placed at different locations near the traffic signal, to guarantee RF signal reception in unfavourable conditions.

Although the experiments described in this communication were carried out using traffic signals, RFID tags can be located in any place on the infrastructure (for example: traffic lights, temporary road diversions, pedestrian crosses, *etc.*). The results suggest that an automatic intelligent speed control system can be used to prevent any unexpected traffic circumstance and improve the safety of the occupants of the vehicle.

## Figures and Tables

**Table 1:** description of the traffic signals used in the circuit of Figure 6. Their position was accurately determined with the GPS system.

**Signal**	**Tag IDs (front, back)**	**UTM coord. (east, north)**	**Information**
1	604123, 604124	459045.89, 4462554.75	Straight road segment, sets target speed to 25 km/h
2	604140, 604148	458974.32, 4462558.7	Curve ahead, sets target speed to 10 km/h
3	604142, 604141	458950.22, 4462543.39	Curve exit, sets target speed to 20 km/h
4	604135, 604127	458948.64, 4462473.12	Curve ahead, sets target speed to 8 km/h
5	604126, 604133	458939.72, 4462465.47	Straight road segment, sets target speed to 25 km/h
